# Comparison of the Ability of High and Low Virulence Strains of Non-cytopathic Bovine Viral Diarrhea Virus-1 to Modulate Expression of Interferon Tau Stimulated Genes in Bovine Endometrium

**DOI:** 10.3389/fvets.2021.659330

**Published:** 2021-04-09

**Authors:** Kai Wang, Carole Thomas, Shujun Zhang, D. Claire Wathes, Zhangrui Cheng

**Affiliations:** ^1^Department of Pathobiology and Population Sciences, Royal Veterinary College, Hatfield, United Kingdom; ^2^Key Laboratory of Agricultural Animal Genetics, Breeding and Reproduction of Ministry of Education, Huazhong Agricultural University, Wuhan, China

**Keywords:** bovine viral diarrhea virus, endometrium, interferon, maternal recognition of pregnancy, virulence, immunosuppression, fertility, interferon stimulated genes

## Abstract

Bovine Viral Diarrhea virus (BVDV) is a pestivirus with a single-stranded, positive sense RNA genome. It is endemic in many cattle populations, causing major economic losses in part due to reduced fertility. BVDV exhibits great genetic diversity and is classified as type 1 or 2 (BVDV-1, BVDV-2) with either non-cytopathogenic (ncp) or cytopathogenic (cp) biotypes. Differing strains of ncpBVDV differ in virulence, affecting clinical outcome. BVDV replicates in the reproductive tract, affecting host immunity and embryo survival. This study used an *in vitro* model of primary bovine endometrial cell cultures to compare the effects of two BVDV ncp type 1a strains of differing virulence (termed HO and KY) on endometrial transcription of candidate interferon stimulated genes (ISG) using qPCR. Half the cultures were stimulated with interferon tau (IFNT, the conceptus produced pregnancy recognition factor) in the presence or absence of viral infection. Cultures were replicated on cells from 10 BVDV-free cows. IFNT treatment stimulated transcription of 10 candidate ISGs, whereas both ncpBVDV-1 strains alone inhibited transcription of 8/10 ISGs. In combined BVDV-1+IFNT cultures, the stimulatory effect of IFNT on expression of *GBP4, ISG15, HERC5, RSAD2, IFIH1, IFIT3*, and *MX1* was significantly inhibited by HO, but only *ISG15, RSAD2, IFI27*, and *IFIT3* were decreased by KY. Inhibition by HO was generally greater. The IFNT-induced expression of *TRIM56* was, however, increased by HO. These data show that HO, the more virulent ncpBVDV-1 strain, has a greater capacity to inhibit key antiviral pathways. These differences need confirmation at the protein level but may influence immune tolerance of the host. They could also reduce fertility by increasing uterine susceptibility to bacterial infection and disrupting IFNT-mediated pregnancy recognition.

## Introduction

Bovine Viral Diarrhea virus (BVDV) is a member of the pestivirus genus of the family *Flaviviridae*. It has a single-stranded (ss), positive sense RNA genome of ~12.3 kb which is able to replicate in many types of tissues including the reproductive tract (1, 2). BVDV is classified as type 1 (BVDV-1) or type 2 (BVDV-2) on the basis of sequence differences within the 5′ untranslated region (UTR). It also has either non-cytopathogenic (ncp) or cytopathogenic (cp) biotypes (3, 4). Both the biotype and the timing of infection have a profound influence on the clinical outcome. Acute infection of cattle with a low virulence ncp biotype causes decreased production, poor fertility, and immunosuppression (5). Infection early in gestation may cause pregnancy loss, whereas transplacental infection of the fetus later in pregnancy may either cause abortion, congenital defects or result in the birth of an immunotolerant calf that is persistently infected with BVDV (1). In contrast, high virulence BVDV-2 isolates cause severe acute infections characterized by pyrexia, profound leucopaenia, thrombocytopaenia, diarrhea, respiratory dysfunction, mucosal erosions, and hemorrhage (3, 6, 7). Some isolates of BVDV-1 also show higher virulence and similarly cause debilitating disease (8–11). BVDV currently remains endemic in many cattle populations worldwide, with its adverse effects having major economic consequences (12).

Poor fertility is also a serious problem impacting the global dairy industry (13, 14). Among many factors contributing to reproductive failure, embryonic death is an important player, affecting up to 40% of all potential pregnancies (15, 16). About 70–80% of embryonic losses occur before day 16 of gestation, resulting in failure of the maternal recognition of pregnancy (MRP) (15, 17, 18). The early bovine embryo can already modulate the composition of the uterine luminal fluid by day 7 after insemination (19). In ruminants the main signal involved in MRP is, however, interferon-τ (IFNT) released by the trophectoderm. The conceptus begins IFNT production at around day 8 of gestation, and this must increase and reach a sufficient threshold level by day 16 to ensure MRP and prevent luteolysis (20–22). In concert with promoting the continued production of luteal progesterone, IFNT acts in a paracrine manner on the uterine endometrium to develop a receptive environment for implantation. This includes causing changes in the production and/or localization of type I interferons, cytokines and prostaglandins (PGs) (23).

Among these factors, type I interferon stimulated genes (ISGs) are amongst those most upregulated by IFNT (21, 24, 25) via the STAT1 and STAT2 - IRF signaling pathway (25). ISGs play crucial roles in the establishment of pregnancy via modulation of uterine immunity, stromal remodeling, stimulating hyperplasia of the endometrial glands, and development of the uterine vasculature (23, 26, 27). Of these, *ISG15* is one of the most highly upregulated genes during implantation in cows. The protein has a dual function: it can either be secreted as a cytokine or act intracellularly as an ubiquitin-like modifier of target proteins through a process known as ISGylation (28). This process is considered part of the maternal response to the developing conceptus, implantation and placentation, which is conserved across diverse mammalian species (humans, ruminants, mice) (29). Although the precise role of ISG15 remains to be determined, its' up-regulation during pregnancy may protect the conceptus against infection and promote placental development and vascularization. In Isg15(–/–) mice the number of implantation sites were similar to the wild type on 7.5 days post coitus (dpc) but embryo mortality increased by 12.5 dpc, resulting in significantly smaller litter sizes by an average of 3.4 pups. In these ISG15 null mice lesions were detected in both antimesometrial decidua and trophoblast cells and there was a reduction of 65% in the migration of natural killer cells into the mesometrial pole (30).

Our previous *in vitro* studies demonstrated that processes vital to the establishment of pregnancy were disrupted via two mechanisms in bovine endometrial cells infected with Pe515nc, a type 1a biotype of ncpBVDV. The first involved modulation of the endometrial PG signaling pathways (31). Secondly, IFNT-induced ISG production by the endometrium was also inhibited (24). This inhibition was achieved, at least in part, by suppressing the activation effect of IFNT on the signaling pathways associated with IFNT receptors, JAK1/TYK2, IRFs, STAT1, and STAT2 (25). There is substantial evidence that infection with BVDV is strongly associated with pregnancy failure (32–35). Reported effects include decreased conception and pregnancy rates, prolonged calving intervals and increased intervals to first calving (36). Furthermore, a meta-analysis of 41 studies showed that in field trials vaccination against BVDV increased pregnancy risk by ~5% (37). It is likely that the mechanisms whereby BVDV can interfere with pregnancy establishment are at least partly responsible for the decline in fertility found in BVDV-infected cattle.

The outcome of any viral infection depends on a viral modification to cellular pathways, leading to avoidance or initiation of innate immune responses and/or apoptosis. With respect to the virulence of different strains of ncpBVDV, experimental studies have established that although the route and initial spread of infection are the same, the amount of viral antigen in tissues and the speed of transmission for high virulence strains rapidly exceed that observed in low virulence strains (38, 39). Infection with ncpBVDV can dampen innate immune responses in several ways. Virus encoded N^pro^ suppresses the type I IFN response by causing proteolysis of IRF3 (40). The secreted structural protein E^rns^ has also been shown to block stimulation of the innate pathways through its extracellular function as a ribonuclease effective against both single (ss) and double (ds) stranded RNA (41). Conversely, cpBVDV strains cause a greater magnitude of enhancement of ISGs and stress inducible genes than ncpBVDV (42). We have, however, been unable to find any previous studies which have compared the mechanisms involved with different virulence between strains of ncpBVDV-1 and none have previously examined such effects in endometrial tissues with respect to the establishment of pregnancy. We hypothesized that BVDV strains with different virulence would differ in their effects on uterine ISG production and how this was modulated by IFNT. Our aims were to compare the effects of two strains of ncpBVDV-1, KY1203 [KY, with low virulence (43)] and Ho916 (HO, with high virulence) (8). Our results demonstrated that both KY and HO inhibited basal and IFNT-induced expression of uterine ISGs, with a stronger effect attributed to the higher virulence strain of HO.

## Materials and Methods

Unless otherwise stated, reagents and consumables were purchased from Sigma-Aldrich (Poole, Dorset, UK) or Thermo Fisher Scientific (Paisley, UK); culture media contained 50,000 units/L penicillin and 50 mg/L streptomycin; cell isolation and culture procedures were carried out under sterile conditions and the cells were cultured at 37°C with 5% CO_2_. All culture media including serum used for cell isolation and culture were certified BVDV-free.

### Animals, Cell Isolation, and Culture

Uteri from cows in the early luteal phase of the oestrous cycle (determined by the presence of a newly formed corpus hemorrhagicum in one of the ovaries) and which appeared healthy on gross examination were collected at the local abattoir (44). Approval of an independent ethical committee was not needed as the reproductive tracts used were a waste product of the regular slaughter process. On return to the laboratory, endometrial samples from all tracts were tested for potential contamination with BVDV by conventional PCR using the primer pairs: forward (5′ ATGCCCWTAGTAGGACTAGCA; position 108 - 128 with respect to NADL genome) and reverse (5′ TCAACTCCATGTGCCATGTAC; position 395–375 with respect to NADL genome) as described below (31, 45). A BVDV-positive control prepared using the pT7Blue-2 blunt vector, linearized (Novagen, Cambridge, MA02139, USA) and a reference gene *ACTB* (see Table 1 for primers) were included in each assay. This BVDV-testing was performed in parallel with the cell isolation. None of the samples tested positive, so all endometrial samples used for cell culture were BVDV-free.

**Table 1:** Primer sequence information for qPCR.

**Gene**	**Primer sequence (5^**′**^-3^**′**^)**	**GenBank accession number**	**Product length (bp)**	**Annealing (^**°**^C)**
**Candidate interferon stimulated genes (ISGs)**				
*ISG15*	F: AGAAGATCAATGTGCCTGCTTT R: CTTGTCGTTCCTCACCAGGAT	NM 174 366.1	161	59.4
*DDX58*	F: ACCCAGTTGTTTCACAGCCA R: GACATGAAAGGCACAGGGGA	XM-002689480.4	137	60.8
*GBP4*	F: GAATCACTGCCTCCTCCTTG R: CCCACCAAGAGTTTGGATGA	NM-001102261.2	197	63
*HERC5*	F: TTCAGACCCCAAATCAGGAC R: TCTGGGCTCTGCTCTCTTTC	NM-001101995.1	193	62
*IFIH1*	F: GGTGATGGAAAGTACGGGCA R: CACCGTTTCCCAGTTGTCCT	XM-010802053.1	194	60.8
*IFI27*	F: GGAACGTGCTGTGCAACTAA R: TTTGTCGAGTGCTTTCATGC	NM-001038050.2	145	58
*IFIT3*	F: TCCGAACCAACAGAGACAGC R: TCTGCCTCTGGTCTGGATCA	NM-001075414	111	58.2
*MX1*	F: TATATGATCGTGAAGTGCCGGG R: AGCTCGGTGGTAAGTCTTTCTG	NM-173940.2	113	58.2
*RSAD2*	F: CCCTGAAAACGCTGGAGGAT R: GGCAGATGGGTCAGTGTCAA	NM-001045941.1	150	61.4
*TRIM56*	F: TTCAGACCCCAAATCAGGAC R: TCTGGGCTCTGCTCTCTTTC	NM_001206574.1	126	62
**Cell markers**				
*KRT18*	F: aagacctgaatgaccgcctg R: tcttcaggtaatgcgcccag	NM_001192095.1	141	61
*VIM*	F: cctggagcagcagaacaaga R: gctttgtcgttggtgagctg	NM_173969.3	138	61
**Candidate reference genes**				
*ACTB*	F: GAAATCGTCCGTGACATCAA R: AGGAAGGAAGGCTGGAAGAG	NM-173979.3	182	59.5
*GAPDH*	F: GGTCACCAGGGCTGCTTTTA R: TTCCCGTTCTCTGCCTTGAC	NM-001034034.2	147	60.2
*RPL19*	F: TCGATGCCGGAAAAACAC R: ATTCTCATCCTCCTCATCCAG	NM-001040516	119	60.2
*18SrRNA*	F: CGGCGACGACCCATTCGAAC R: GAATCGAACCCTGATTCCCCGTC	AY779625	99	64.5

A mixture of primary epithelial and stromal cells from 10 mature cows was isolated and cultured following the methods described previously (24, 31). Briefly, strips of intercaruncular endometrium were dissected and put into Dulbecco's Modified Eagle's Medium/Nutrient Mixture F-12 Ham (DMEM/F12 medium) (Sigma). Using a mechanical tissue chopper (McIIwain Laboratory Engineering, Guilford, Surrey, UK), the strips were chopped into about 1 mm^3^ cubes and chopped tissue was placed into two 50 mL sterile vials (20 g each). The vials were mixed with 30 mL digestive solution containing 100 mg bovine serum albumin (BSA, Sigma), 50 mg trypsin III (Worthington, Lakewood, NJ 0 8701, USA), and 50 mg collagenase A (Roche, Welwyn Garden City, UK) per 100 mL of Hanks balanced salt solution (HBSS; Sigma) and incubated for 90 min at 37°C with 5% CO_2_ and manual mixing every 30 min. After this, the cell suspension was filtered through a 100 μm mesh into 50 mL falcon vials and washed two times using HBSS containing 10% FBS and 3 μg/mL trypsin inhibitor (Sigma) and centrifuged at 100 × g and 10 °C for 10 min. Then the cells were suspended with culture medium (DMEM/F12 medium with 10% FBS) and plated in 24-well IWAKI micro plates (Scitech DIV, Asahi Techno Glass, Japan) at 2 mL per well containing 0.5 × 10^5^ cells. This was considered as day 1. The culture medium was changed every 48 h.

### Infection of Bovine Endometrial Cells With ncpBVDV-1 and IFNT Treatment

Two type 1a ncpBVDV strains KY1203 (KY) and Ho916 (HO) both isolated in the UK were used for this study. KY1203 is a strain with low virulence (43). Ho916 was a high virulence strain isolated from an acute outbreak that resulted in 50% herd mortality in adult cattle (8). Inoculation of endometrial cultures was carried out following methods validated in our laboratory (24).

Virus derived from early passages from the original field isolates of Ho916 or from the sub-master stock of KY1203 were used in the procedures. They were not therefore adapted to cell culture so should have maintained their original characteristics at the time of use. Stocks were amplified, maintained and titrated in BVDV-free Madin-Darby bovine kidney (MDBK) epithelial cells (American Type Culture Collection) as previously described (46). For the TCID_50_ assay virus was titrated by end point dilution assay. Following 5 days incubation cells were fixed and virus detected by immunostaining using anti-BVDV hyper-immune serum at 1:200 dilution in 5% normal rabbit serum in PBS containing 0.05% Tween-20 (PBS-T). After incubation for 45 min at 37°C the cells were washed (4 times PBS-T). Samples were incubated with the appropriate secondary antibodies (rabbit anti-bovine IgG alkaline phosphatase conjugate diluted 1:1,000) for 45 min at 37°C. Following final washes and addition of BCIP®/NBT substrate solution (ThermoFisher Scientific), virus positive wells (as identified by immunostaining) were scored. This assessment procedure was used rather than cytopathic changes, as both strains were classified as being ncp. Virus titer was calculated as 50% Tissue Culture Infective Dose per ml (TCID50/ml) using the Spearman-Karber equation. The multiplicity of infection (MOI) was calculated based on the TCID_50_ quantification.

Inoculation of endometrial cultures was carried out following methods validated in our laboratory (24). Cells from each cow were grown in three separate 24-well plates to prevent cross-contamination between each BVDV strain and the non-infected controls. The treatment times and doses for ncpBVDV-1 infection were optimized previously (46). Inoculation was performed on day 4 of culture. Cells were infected with the appropriate BVDV strain at a MOI of 0.1, or an equivalent volume of media for uninfected controls. On day 8, 4 days after the BVDV infection, half the wells received medium containing 100 ng/mL IFNT (recombinant ovine IFNT, Cell Sciences, Canton, USA) and incubation continued for a further 24 h. The cells from every cow, therefore, received six treatments designated as Control (CONT), IFNT, KY, HO, KY+IFNT, and HO+IFNT, each of which was replicated in 4 wells. At the end of culture (day 9), the cells from each treatment group in each cow (4 wells) were pooled for total RNA extraction using RNeasy Mini kits (Qiagen, Manchester, UK) following the supplier's protocol and stored at −80°C for PCR and qPCR assays.

### Assessment of BVDV Cell Infection and Cell Viability

Both a PCR method and an indirect enzyme (alkaline phosphatase) immunostaining procedure were used to confirm bovine endometrial cell infection with ncpBVDV-1 (46). The cell viability after exposure to the infection and treatment was assessed using an MTS reduction assay method (CellTiter 96 AQueous One Solution Cell Proliferation Assay, Promega Corporation, Southampton, UK) as described previously (44). Immunocytochemical staining against CD172 (anti-CD172a, DH59B; Monoclonal Antibody Center VM&P, Washington State University) validated in our laboratory was used to determine potential contamination of the endometrial cells with immune cells (44).

### Primer Design

The primers for both conventional PCR and real time PCR (qPCR) were designed using DNA sequences obtained from GenBank (https://www.ncbi.nlm.nih.gov/gene/) and a Primer3 online version 4.1.0 (http://bioinfo.ut.ee/primer3/). According to the recommendation by PCR Biosystems (London, UK) who supplied the reagents for cDNA synthesis (RT) and qPCR, the amplicon length was optimized as 100–200 bp and the melting temperature was around 60 °C. The alignment specificity and quality were confirmed with the Blast tool (https://www.ncbi.nlm.nih.gov/tools/primer-blast/). Information on primer sequences is given in Table 1. Designed primers were made by Eurofins Genomics (Ebersberg, Germany).

### PCR and qPCR

To prepare the RNA sample for both PCR and qPCR, potential genomic DNA contamination in the RNA extract was eliminated using a DNase I kit (Thermo Scientific) based on the supplied protocol. The treated RNA (1 μg for each sample) was reverse transcribed into cDNA using a cDNA synthesis kit (PCRBiosystems, London, UK) following the method described previously (24). The resulting cDNA was diluted to 100 μL and stored at −80°C until use. To minimize potential variation, a mastermix of reagents was made and all samples were run under the same conditions.

Conventional PCR was used to check the specificity of the primers and to produce the gene amplicons for preparing standard curves used in the absolute qPCR and carried out according to the method and reagents described previously (24) and the primers listed in Table 1. After the specificity of primers was verified using electrophoresis on a 2% (w/v) agarose gel, the cDNA amplicon for each gene was purified using a QIAquick PCR purification kit (Qiagen). The quality and concentrations were determined with a DeNovix DS-11 spectrophotometer (DeNovix Inc., Wilmington, USA). These were stored at −80°C for subsequent use in the qPCR standard curve and annealing temperature optimization.

The expression of four potential reference genes (*GAPDH, RPL19, ACTB*, and *18SrRNA*), two cell type markers (*KRT19* and *VIM*) and 10 candidate genes (*ISG15, DDX5, GBP4, HERC5, IFIH1, ITIT3, IFI27, MX1, RSAD2, TRIM56)* were quantified with absolute qPCR using the methods described previously (24). To optimize the annealing temperature which produced maximal amplification and determine the amplicon-specific melting temperatures of the primers, a temperature gradient (55–65°C) qPCR with 8 identical reactions was carried out using a gradient function of the qPCR machine (CFX96 Real-Time System DNA, Bio-Rad Laboratories, CA, USA). Each reaction contained 2 ng of the DNA standard, 10 μl Sygreen Mix (PCR Biosystems), 0.8 μl of each 10 μM forward and reverse primer, and nuclease-free water added up to 20 μl. Each qPCR assay contained a standard curve with eight concentrations ranging from 1 to 1 × 10^−7^ ng/mL, no template control (NTC) and sample cDNA from RT. To minimize variation all these were prepared in duplicate with the same mastermix of reagents. Each qPCR vial contained 5 μl of cDNA standard (purified) or samples (cDNA sample), 10 μl Sygreen Mix (PCR Biosystems), 0.8 μl of 10 μM forward primer, 0.8 μl reverse primer (see Table 1), and 3.4 μl nuclease-free water. The qPCR assay was performed in a Bio-Rad CFX Real-Time qPCR system (Bio-Rad, Hercules, USA). The thermal cycle programme included a Tag activation at 95 °C for 2 min, 38 cycles of denaturing at 95°C, and annealing/extension for 30 s at the optimized temperatures listed in Table 1. A melting curve analysis was included to check the quality of the qPCR assays. The results were analyzed using the CFX Manager Software package (Bio-Rad). The limit of quantification was 1 × 10^−6^ to 1 × 10^−7^ ng/mL for all tested genes. The expression values of mRNA were initially quantified as fg/μg RNA by the absolute qPCR.

### Statistical Data Analysis

Stability of the reference genes (*ACTB, GAPDK, RPL19*, and *18SrRNA*) was tested using GeNorm software (Ghent University Hospital Center for Medical Genetics, Belgium). Genes with small M values were considered as stable genes. The effect of treatment groups on gene expression was also determined with analysis of variance (ANOVA) built in SPSS V26 (Chicago, IL, USA). As the results showed that *ACTB* and *RPL19* were stable (Supplementary Figure 1), the expression values of all target genes were normalized to the expression values of the geometric mean of *ACTB* and *RPL19*. Differences of the normalized values for each candidate gene were identified using ANOVA via a linear mixed effect model built in SPSS 26, in which treatment (CONT, KY, HO, IFNT, KY+IFNT, and HO+IFNT) was taken as fixed effect and cow (batch) as random effect with covariance type of variance components, and restricted maximum likelihood was used for estimation. The homogeneity of variance across the groups was analyzed with Levene's test. Logarithmic transformation was carried out for the genes whose expression was not homogeneous (*HERC5* and *IFI27* only). Significance was considered at *P* < 0.05. Where ANOVA showed significant differences between treatment groups, Fisher's Least Significant Difference (LSD) multiple comparisons were used to compare the differences between each treatment pair.

## Results

### Validation of Cell Culture and ncpBVDV-1 Infection

**Figure 1:**
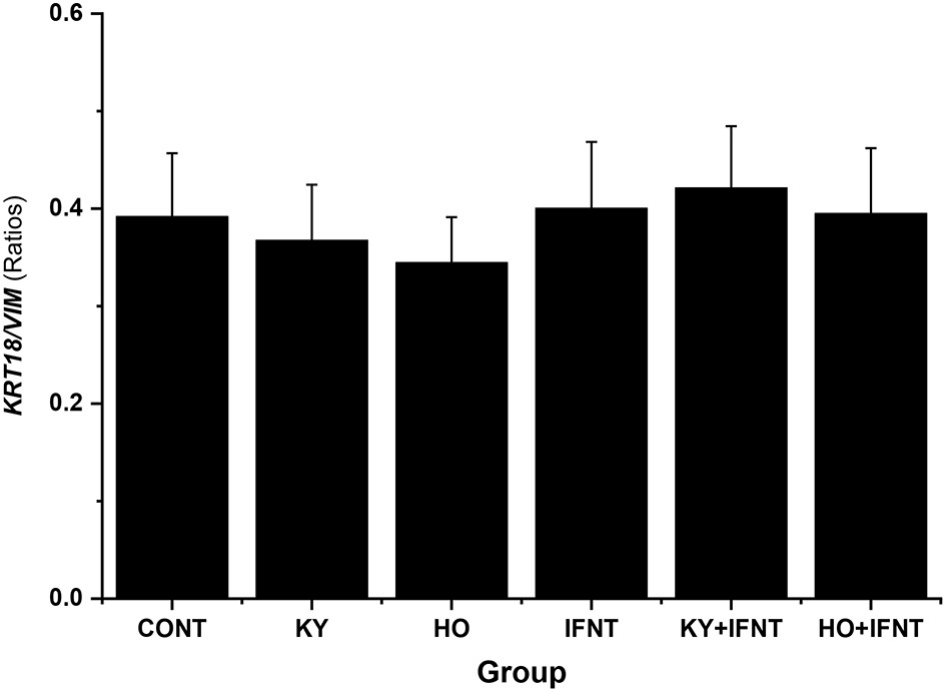
Effect of two ncpBVDV-1 strains, IFNT and their combination on the ratio of epithelial to stromal cells present in the cultures. Primary cultures of mixed bovine endometrial cells (epithelium plus stroma) were cultured for 4 days before inoculation with either KY1203 (KY) or Ho916 (HO) strains of ncpBVDV-1. After a further 4 days, half the cultures were then stimulated with 100 ng/mlL IFNT and the cultures were terminated 24 h later. CONT, untreated control cultures. All treatments were replicated using cells from 10 cows. Values are mean ± SEM. There was no difference in ratio due to treatment (ANOVA, *P* > 0.05). Gene expression of *KRT18* and *VIM* was used as markers of epithelial and stromal cells, respectively.

**Figure 2:**
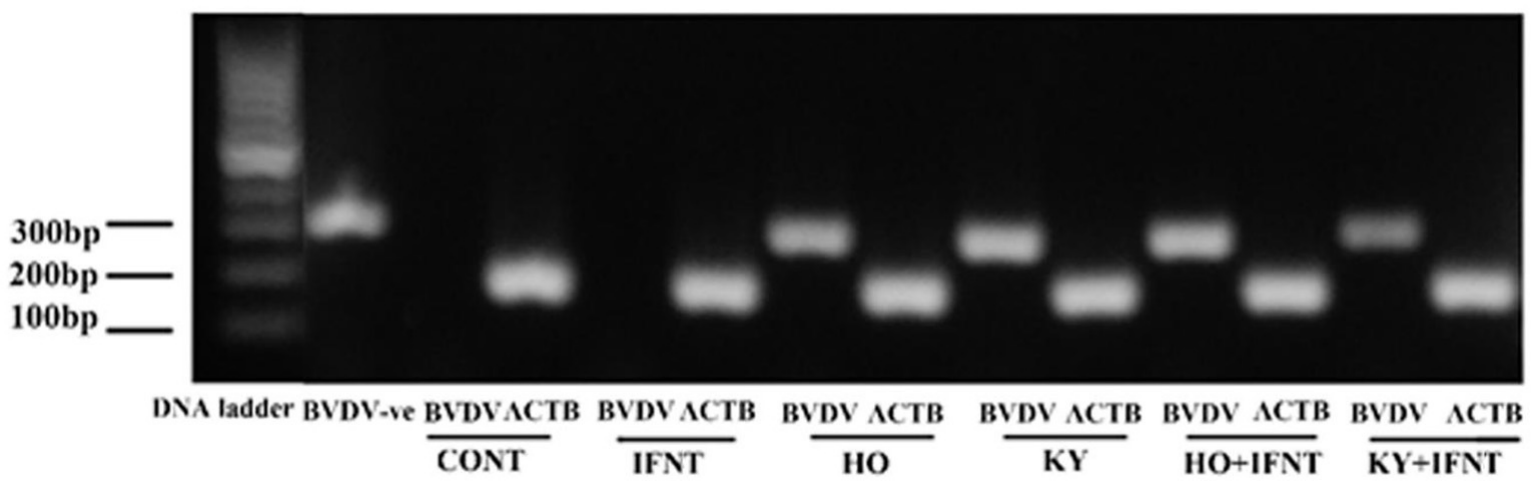
PCR validation demonstrating infection of primary cultures of mixed bovine endometrial cells (epithelium plus stroma) with either Ho916 (HO) or KY1203 (KY) strains of ncpBVDV-1. Cells were cultured for 4 days before inoculation with each BVDV strain at a multiplicity of infection (MOI) of 0.1. After a further 4 days, half the cultures were then stimulated with 100 ng/mL IFNT and the cultures were terminated 24 h later. The expected product sizes for BVDV and the β-actin gene (ACTB) are 288 and 182 bp, respectively. The first two lanes contain BVDV-positive and negative controls.

The MTS reduction assay illustrated that treatments did not alter cell viability significantly (data not shown). The expression values of keratin 18 (*KRT18*) and vimentin *(VIM)* were used as markers for epithelial and stromal cells, respectively. At the end of culture these two cell types were present at a ratio of about 0.4 and no treatments altered this ratio (*P* > 0.05) (Figure 1). Contamination of immune cells in the cultures was negligible (<0.001%). Immunocytochemical staining showed that both the high and low virulence strains of ncpBVDV-1 (HO and KY) infected the endometrial cells successfully and that they proliferated in the 5 days after inoculation (Supplementary Figures 2A,B) but no cross contamination occurred with the uninfected control cultures (Supplementary Figures 2C,D). PCR analysis confirmed that the endometrial cells were infected by both ncpBVDV-1 strains in the presence or absence of IFNT treatment (Figure 2). Overall these results confirmed the validity of the cell culture model by showing that there were consistent populations of mixed endometrial epithelial and stromal cells in our system and that neither ncpBVDV-1 strains nor IFNT affected their growth or viability.

### Effect of ncpBVDV-1, IFNT, and Their Combination on Expression of the Selected Reference Genes by Uterine Endometrial Cells

Four potential reference genes (*ACTB, GAPDH, RPL19*, and *18SrRNA*) were quantified in the cultured endometrial cells using qPCR and all were well expressed. Analysis using GeNorm showed that both *GAPDH* and *18SrRNA* had large M values (>1) and ANOVA confirmed that the treatments altered expression of both *GAPDH* and *18SrRNA* (*P* < 0.01), whereas expression of *ACTB* and *RPL19* had small M values (<1) and was not significantly affected by any treatment (*P* > 0.05) (Supplementary Figure 1). The geometric mean of *ACTB* and *RPL19* was, therefore, used for normalization and subsequent analysis was carried out using the normalized values.

### Effect of ncpBVDV-1 Alone on Expression of Basal ISGs by Uterine Endometrial Cells

**Figure 3:**
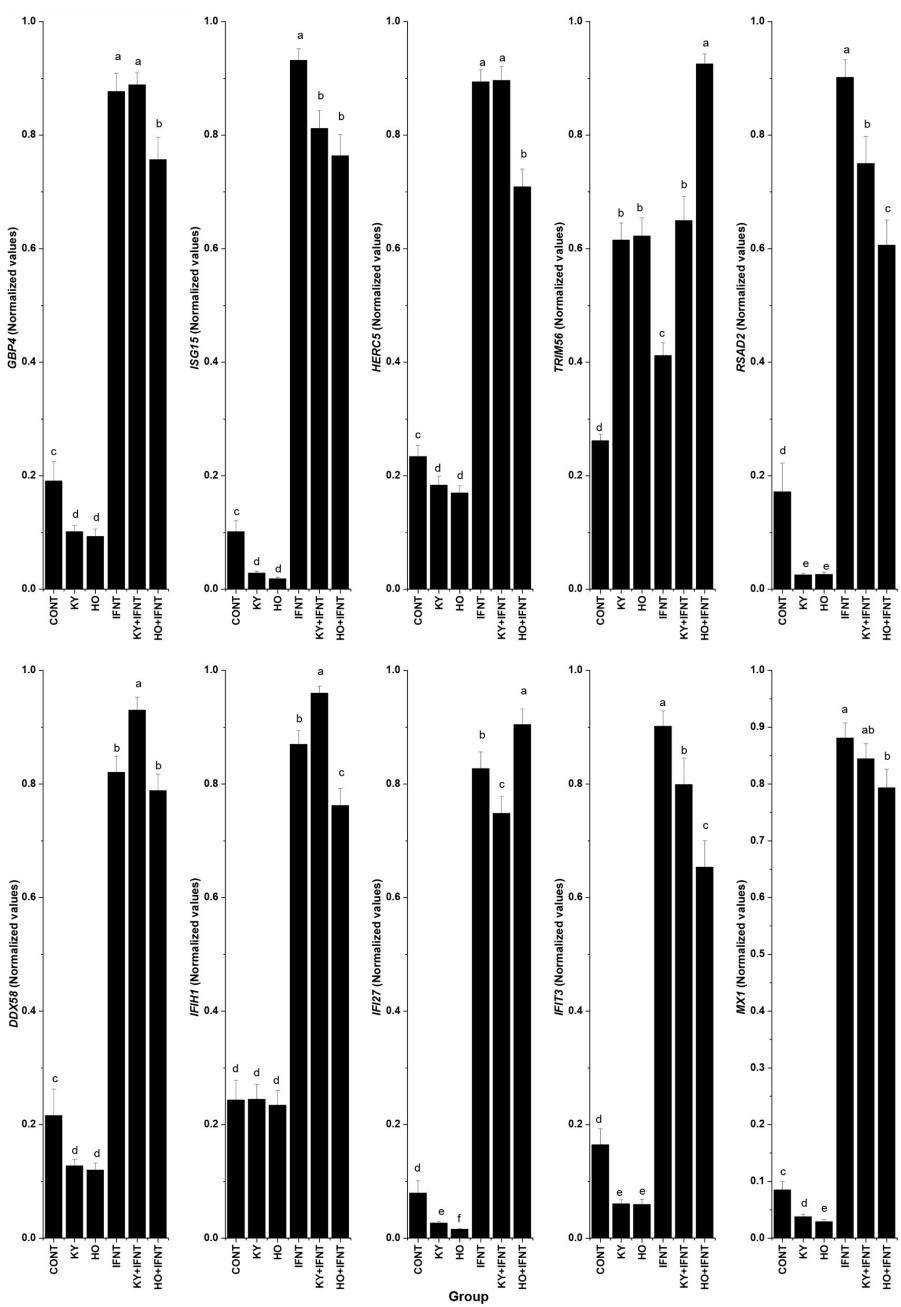
Effect of two ncpBVDV-1 strains, IFNT and their combination on the expression of 10 candidate genes. Gene expression was measured using qPCR and normalized using *ACTB* and *RPL19* as reference genes. Primary cultures of mixed bovine endometrial cells (epithelium plus stroma) were cultured for 4 days before inoculation with either KY1203 (KY) or Ho916 (HO) strains of ncpBVDV-1. After a further 4 days, half the cultures were then stimulated with 100 ng/mL IFNT and the cultures were terminated 24 h later. CONT, untreated control cultures. All treatments were replicated using cells from 10 cows. Values are mean ± SEM, a>b>c>d>e, *P* < 0.05– <0.001.

Ten ISGs were tested for the effect of the two ncpBVDV-1 strains and the results are given in Figure 3. In the endometrial cells infected with either KY or HO, expression of eight ISGs (*GBP4, ISG15, HERC5, RSAD2, DDX58, IFI27, IFIT3*, and *MX1*) was significantly inhibited (*P* < 0.05–0.01). The high virulence strain HO produced more reduction of *IFI27* and *MX1* expression than the low virulence strain KY (*P* < 0.05). In contrast, expression of *TRIM56* was significantly stimulated by infection with both ncpBVDV strains (*P* < 0.01). Neither KY nor HO strains altered the expression of *IFIH1* (*P* > 0.05).

### Effect of IFNT on Expression of Candidate Genes in Endometrial Cells With or Without ncpBVDV-1 Infection

IFNT treatment alone intensively increased expression of all tested ISGs compared with the CONT (*P* < 0.05–0.001). In the presence of HO infection, the stimulatory effect of IFNT on expression of *GBP4, ISG15, HERC5, RSAD2, IFIH1, IFIT3*, and *MX1* was significantly reduced (IFNT vs. IFNT+HO, *P* < 0.05–0.01). In cells infected with the KY strain, the stimulatory effect of IFNT on expression of *ISG15, RSAD2, IFI27*, and *IFIT3* was significantly decreased (IFNT vs. IFNT+KY, *P* < 0.05). Compared with the KY+IFNT group, the HO+IFNT group had significantly lower expression of *GBP4, HERC5, RSAD2, DDX58, IFIH1*, and *IFIT3* (*P* < 0.05–0.01), but significantly higher expression of *TRIM56* and *IFI27* (*P* < 0.05). The IFNT induced expression of *TRIM56, DDX58*, and *IFIH1* was further enhanced in the cells infected with KY (IFNT vs. IFNT+KY, *P* < 0.05–0.01) and that of *TRIM56* and *IFI27* was further increased in the presence of HO infection (IFNT vs. IFNT+HO, *P* < 0.05) (Figure 3). These results are summarized in Table 2.

**Table 2:** Summary comparison of the effects of KY1203 (KY) and Ho916 (HO) strains of ncpBVD-1 on gene expression by cultured bovine uterine endometrial cells, with or without IFNT stimulation.

**Gene**	**KY v CONT**	**HO v CONT**	**IFNT v CONT**	**KY+IFNT v IFNT**	**HO+IFNT v IFNT**	**Comparison KY and HO^a^**
ISG15	↓	↓	↑	↓	↓	same
IFIT3	↓	↓	↑	↓	↓↓	^*^
RSAD2	↓	↓	↑	↓	↓↓	^*^
MX1	↓	↓↓	↑	(↓)	↓↓	^*^
GBP4	↓	↓	↑	=	↓	^*^
HERC5	↓	↓	↑	=	↓	^*^
DDX58	↓	↓	↑	↑	=	^†^
IFI27	↓	↓↓	↑	↓	↑	^‡^
IFIH1	=	=	↑	↑	↓	^‡^
TRIM56	↑	↑	↑	↑	↑↑	^*^

a *Shading has been used to highlight differences between the two ncpBVDV-1 strains: = no change in expression compared with the control cultures, ↑ increased expression, ↓ decreased expression, (↓) trend to reduced expression*.

* *Greater effect of HO*;

† *Greater effect of KY*;

**Figure 4:**
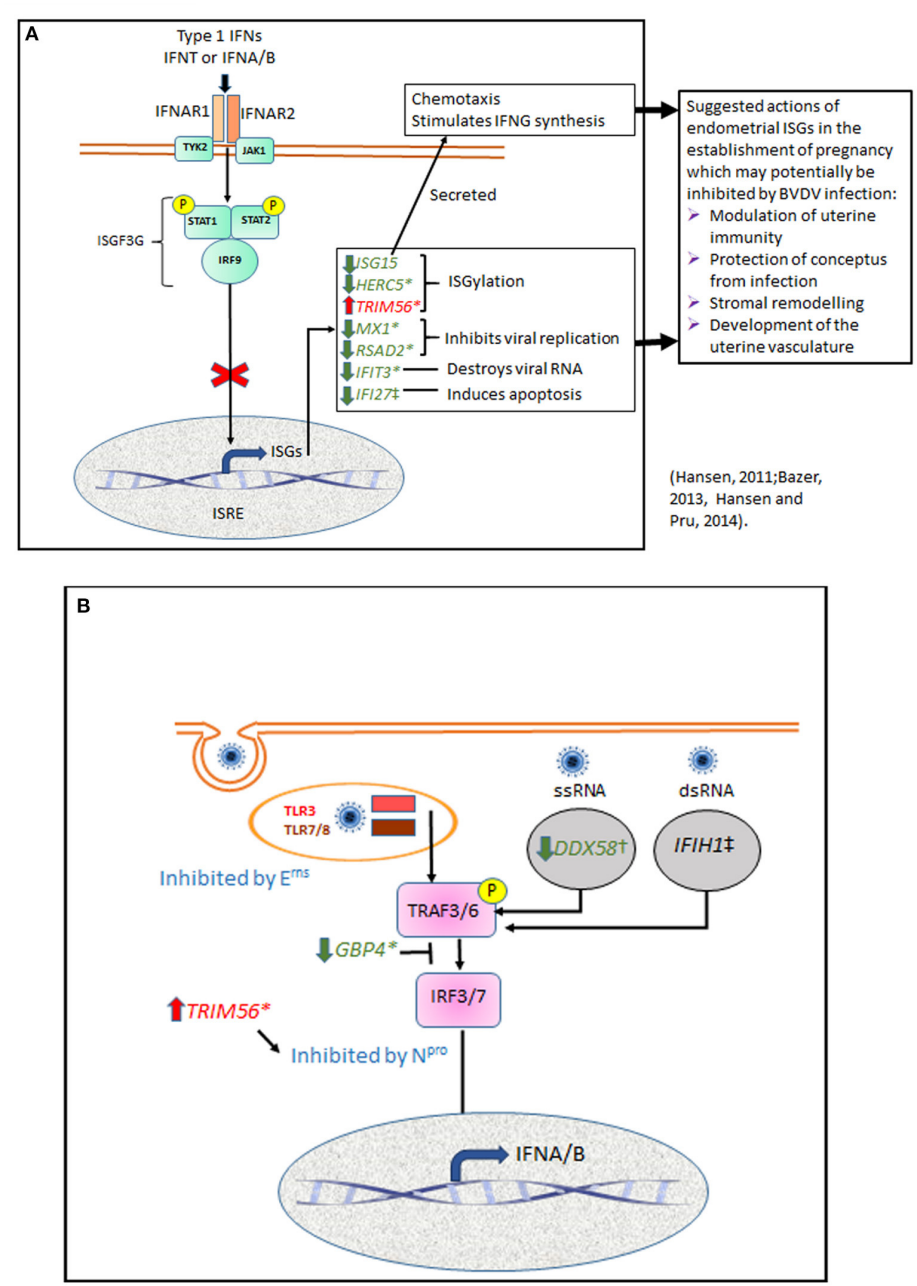
Summary diagram showing potential points in endometrial cell signaling pathways which may be influenced by BVDV infection. **(A)** Many cell types respond to viral infection by production of type 1 interferons. IFNA and IFNB have similar structure and biological actions to the bovine pregnancy recognition factor IFNT. All three type 1 IFNs bind to interferon alpha and beta receptor subunits 1 and 2 (IFNAR1 and IFNAR2) to initiate cell signaling via the Janus activated kinases (JAKs) and tyrosine kinase 2 (TYK2) pathways. This phosphorylates STAT1 and STAT2, which then dimerise and combine with interferon regulatory factor 9 (IRF9) to form interferon stimulatory gene factor 3 gamma (ISGF3G) which translocates to the nucleus and activates interferon stimulated response elements (ISRE) to induce transcription of ISGs. We showed previously that ncpBVDV-1 infection of endometrial cells inhibited transcription of *IFNAR1, IFNAR2, TYK2, STAT1, STAT2*, and *IRF9* (25). Inhibition of this pathway will reduce transcription of most ISGs, as confirmed in the present study. ISGs shown in green were inhibited by ncpBVD-1 whereas *TRIM56*, shown in red, was stimulated. Possible effects of these changes in gene transcription on processes important for placental establishment are suggested (27, 29). **(B)** In order to infect cells, BVDV is taken up by endocytosis and incorporated into the endosome, where it may be sensed by TLR3 or TLR7/8. For replication to occur, viral RNA is released into the cytoplasm where it can be detected by the pattern recognition receptors DDX58, which recognizes ssRNA, and IFIH1, which targets dsRNA during the RNA replication phase. TLR and PRR then signal via TNF receptor associated factors 3 and 6 (TRAF3, TRAF6) and IRF3 or IRF7 to increase transcription of type 1 IFN. Virus encoded N^pro^ suppresses the type I IFN response by causing proteolysis of IRF3. TRIM56 is an E3 ligase which interacts with N^pro^ to promote its anti-viral activity. E^rns^ is a viral envelope glycoprotein with ribonuclease activity which can degrade viral RNA mainly in endosomal compartments, so preventing activation of the TLR3 and TLR7 signaling pathways. GBP4 is an IFN-induced GTPase which can disrupt TRAF6 mediated polyubiquitination of IRF7, a prerequisite for IRF7 activation (50). We show here that basal expression of *DDX58* and *GBP4* was inhibited and *TRIM56* expression was stimulated by both KY and HO, but only HO was able to inhibit IFNT-stimulated transcription of *GBP4* and *IFIH1*.

‡ *Opposite effect of HO and KY. See Figure 4 for details*.

## Discussion

In ruminants, IFNT released from the trophectoderm in early pregnancy initiates MRP, through maintenance of the corpus luteum and development of a receptive environment in the uterus for successful implantation (23, 26, 47). IFNT-induced upregulation of uterine ISGs is a vital component of this process (23). Our previous studies using Pe515nc, another ncpBVDV-1 strain, showed that BVDV infection significantly inhibited the stimulatory effect of IFNT on bovine uterine endometrial ISG production (24). We have now confirmed this by showing that infection with low or high virulence strains of ncpBVDV-1 not only inhibited basal production (ncpBVDV-1 alone), but also IFNT-induced upregulation of many ISGs (ncpBVDV-1 + IFNT). HO, the more virulent strain, generally had a greater effect than KY. BVDV infection is well-known to cause systemic immunosuppression (48). This ability not only facilitates survival of the virus within the uterine environment but may well-contribute to the reduced fertility reported in BVDV infected cows.

Binding of IFNT to the bovine endometrial epithelium activates a similar pathway to that used by other Type 1 interferons (IFNA and IFNB) (49) (Figure 4A). They all bind to interferon alpha and beta receptor subunits 1 and 2 (IFNAR1 and IFNAR2) to initiate cell signaling via the Janus activated kinases (JAKs) and tyrosine kinase 2 (TYK2) pathways (51, 52). Receptor binding is followed by phosphorylation of STAT1 and STAT2, which then dimerise and combine with interferon regulatory factor 9 (IRF9) to form interferon stimulatory gene factor 3 gamma (ISGF3G). ISGF3G then moves to the nucleus and activates interferon stimulated response elements (ISREs) to induce transcription of ISGs. Our previous study showed that ncpBVDV infection of endometrial cells with Pe515nc inhibited various steps of this pathway by suppressing IFNT-induced activation of the key genes *IFNAR1, IFNAR2, TYK2, STAT1, STAT2*, and *IRF9* (25). Inhibition of this pathway will therefore reduce ISG production. If cows become infected during early pregnancy, inhibition of IFNT signaling is likely to disrupt the successful establishment of pregnancy.

Previous studies in various cell types have provided evidence that BVDV can evade the host immune response via a number of mechanisms (48). Following attachment to the host cell membrane, the virus is taken up by endocytosis and incorporated into the endosome, where it may be sensed by TLR3 or TLR7/8 (Figure 4B). For replication to occur, viral RNA then needs to be released into the cytoplasm and translated into a single polyprotein which is cleaved into structural proteins (three envelopes and one capsid protein) and about eight non-structural proteins, which are involved in replication and assembly. While in the cytoplasm the viral RNA can be detected by two DEAD box protein pattern recognition receptors (PRR), DDX58 (RIGI) and IFIH1 (MDA5). DDX58 recognizes ssRNA whereas IFIH1 targets dsRNA (53). As BVDV is a single stranded virus, it can only be recognized by IFIH1 during the RNA replication phase (54–56). These various host receptors then signal via TNF receptor associated factors 3 and 6 (TRAF3, TRAF6) and IRF3 or IRF7 to increase transcription of type 1 IFN (40). We showed previously that ncpBVDV could inhibit the endometrial expression of *DDX58, IFIH1*, and *IRF7* (24, 25). Virus encoded N^pro^ also suppresses the type I IFN response by causing proteolysis of IRF3 (40, 57). E^rns^ is a viral envelope glycoprotein with intrinsic ribonuclease activity which inhibits IFN expression induced by both single (ss) and double (ds) stranded RNA. It is thought to act as a decoy receptor which degrades viral RNA mainly in endolysosomal compartments, so preventing activation of the TLR3 and TLR7 signaling pathways in order to maintain a state of innate immunotolerance (41, 58). GBP4 is an IFN-induced GTPase which also reduces virus mediated induction of type 1 IFNs by disrupting the TRAF6 mediated polyubiquitination of IRF7, which is a prerequisite for IRF7 activation (50).

In response to a viral attack, the body mobilizes its' defense systems to restrict, neutralize and remove the virus by activating many effector pathways of IFN-mediated antiviral responses (59, 60) (Figure 4A). Our present study confirmed our previous reports by demonstrating that IFNT challenge activated these same pathways to develop a pro-immune and antiviral environment in the uterus. ISG15 is one of the most upregulated genes in both endometrial cells challenged *in vitro* with IFNT (24) and during pregnancy recognition in cows (21). Its' upregulation in the endometrium may facilitate successful conceptus attachment and act as a defense strategy against infection (21, 29, 47). In the cow both intracellular ISG15 and its conjugates were detected by western blotting on d 17 of pregnancy, with concentrations peaking between days 18 to 23 and then declining to low but detectable levels by d 45 (61). In both cattle and sheep ISG15 was present throughout the endometrium during the period of placental attachment, with heaviest staining in the sublumenal stratum compactum and the glandular epithelium (61, 62). ISG15 mRNA and protein expression were also increased in the bovine corpus luteum in early pregnancy (63), where it may act to increase luteal resistance to luteolysis (27).

ISG15 can be secreted as a cytokine which acts as a chemotactic factor for neutrophils, induces natural killer cell proliferation and stimulates IFN-γ production (28). ISG15 also acts intracellularly as an ubiquitin-like modifier of many target proteins via ISGylation, a process in which the C-terminus of ISG15 is conjugated to lysine residues in the target protein following consecutive catalysis with three enzymes E1, E2, and E3 (64). HERC5 and TRIM56 are both E3 ligases. HERC5 blocks the IFN-mediated rise in the total level of ISGylated cellular proteins via non-specific substrate binding (64). TRIM56 interacts specifically with N^pro^, the N-terminal protease of BVDV, to promote its anti-viral activity (65). In contrast to most other ISGs, *TRIM56* was upregulated by all three strains of ncpBVD-1 investigated (pec515nc, KY and HO) (24; present paper), and this will also contribute to blocking type 1 IFN synthesis.

Others of the ISG genes tested (*MX1, RSAD2, IFIT3, IFI27*) are also part of the host antiviral immune defense whose expression was inhibited by ncpBVDV-1 in endometrial tissues [(24) and present paper]. MX1, together with MX2, are Mx dynamin-like GTPases, which can disturb the transport, transcription and translation of viruses within cells (59, 66). RSAD2 (viperin) inhibits viral RNA replication by interacting with non-structural viral proteins and it can also reduce viral spread by inhibiting the release of newly formed virons from cells (67, 68). *IFIT3* (also called retinoic acid-induced gene G protein, RIGG) encodes a protein which can form a cytoplasmic complex to recognize and destroy viral RNA (69, 70). IFI27 (ISG12) is involved in promoting apoptosis of infected cells via mitochondrial membrane destabilization (71). Expression of many ISGs is also induced in bovine endometrium in response to stimulation with lipopolysaccharide (LPS) (44). This implies that they are also used in immune defense against bacterial infection. Uterine disease after calving is present in about 40% of all dairy cows, and is well-established to be a contributory factor to subsequently reduced fertility (72, 73). LPS-stimulated ISG expression by endometrial tissue was similarly inhibited by infecting the cells with the ncpBVDV-1 strain Pe515nc (46). Cows experiencing immunosuppression due to an ongoing BVDV infection are thus likely to become more susceptible to uterine disease.

A key aim of the present study was to compare the mechanisms of action of two ncpBVDV-1a strains with differing virulence, high (Ho916) or low (Ky1203), either alone or combined with IFNT stimulation. BVDV exhibits great genetic diversity among isolates, contributing to major differences in their virulence (1). Low virulent strains cause minor adverse clinical effects, facilitating host survival and enabling a prolonged period of viral shedding. More virulent BVDV-2 biotypes may, however, have evolved improved mechanisms to replicate within the host. Although this will cause greater pathology, the virus can then be released from the host in higher numbers so improving the chance of viral transmission, and giving the more virulent strain a competitive advantage over a less virulent one. Some previous studies have compared the effects of more or less virulent biotypes of ncpBVDV-1 and−2 both *in vivo* by inoculating calves (39, 74, 75) and *in vitro* using monocyte-derived macrophages (76). The latter study reported that macrophages infected with the highly virulent ncpBVDV-2 strain 1,373 showed reduced phagocytosis, bactericidal activity and downregulated MHC II and CD14 expression. There was, however, no significant difference in their mRNA expression values for a variety of cytokines (*IL1B, IL-4, IL-6, IL-8, IL-10, IL-12, TNFA, IFNG, IFNA, IFNB*, and *TGFB*). To our knowledge there are no data currently available to compare the effects of ncpBVDV-1 strains with differing virulence on the host transcriptome, and most previous *in vitro* studies into the effects of BVDV on gene transcription have utilized immune cells [e.g., (77)] or MDBK cells [e.g., (42, 78)] rather than cells derived from the reproductive tract. Previous work has, however, provided evidence that different cell types differ in their responses to ncpBVDV (42).

In our comparison of the effects of HO and KY on endometrial cells, out of all the ISGs tested only *ISG15* gave very similar responses to all treatments (Table 2). Infection with either HO or KY alone had similar inhibitory effects on most ISGs (*ISG15, GBP4, HERC5, RSAD2, DDX58*, and *IFIT3*), although a slightly greater inhibitory effect of HO was observed on *IFI27* and *MX1* (Figure 4). Neither BVDV strain alone altered the expression of *IFIH1*. In contrast, most of the responses in the IFNT-challenged cells differed between the two viral strains. The stimulatory effect of IFNT on *RSAD2, IFIT3* and *MX1* was inhibited to a greater extent by HO infection and only HO inhibited *GBP4* and *HERC5* expression. For *DDX58* there was a synergistic effect whereby gene expression in endometrial cells was increased by the combination of IFNT and KY, whereas there was no additional effect of HO above IFNT alone. For a further two genes, the two BVDV strains had opposite effects on IFNT-stimulated expression: *IFIH1* was increased by KY + IFNT but decreased by HO + IFN but for *IFI27* the reverse was true. Both ncpBVDV strains either alone or combined with IFNT significantly induced expression of *TRIM56* and IFNT challenge enhanced the effect of HO, but not KY on its expression. Liu et al. (78) observed previously that infection of MDBK cells with a type 1b ncp strain of BVDV (BJ-2016, derived from commercial bovine fetal serum) caused downregulation of *ISG15, MX1*, and *OSA1Y* together with a number of genes which form part of the complement and coagulation signaling cascades. This suggested that these pathways may contribute to the inhibition of the host innate immune system during BVDV infection. Further studies are required to determine whether the differential expression of ISGs which we observed between type 1a strains are also important in determining virulence of the virus in host tissues.

It is important to note that differences in transcript were only investigated at a single time point, 5 days following BVDV infection and 24 h after IFNT challenge. The time courses of response may differ between high and low virulence strains. The study is only based on gene transcription and the viral infection is also likely to influence gene translation and processing of the proteins produced. We did, however, confirm in our earlier work that *ISG15* expression in bovine endometrial cultures was reflected in secreted protein levels (24) and a number of other studies have confirmed the presence of protein in the bovine endometrium, which peaks between days 18–23 of pregnancy (61) when placental attachment is initiated (79). The data therefore provide some novel insights into how endometrial tissue may react to BVDV infection. It was clear from our results that the more virulent strain was able to manipulate ISG gene transcription to a greater extent and thus to induce changes which would help the virus to evade initial detection (greater stimulation of *TRIM56* and inhibition of *IFIH1*). If these changes in transcription were reflected in protein concentrations, then many host proteins produced in response to type 1 IFN stimulation, and which can inhibit viral replication, would be suppressed. These included *HERC5, MX1, RSAD2*, and *IFIT3*. The different strains had opposing effects on IFNT stimulated expression of two ISGs, *IFIH1* (increased by KY, inhibited by HO) and *IFI27* (increased by HO, inhibited by KY). *IFIH1* encodes a PRR which targets dsRNA and can therefore only recognize ncpBVDV-1 during the RNA replication phase, again suggesting that endometrial cells may be able to mount a greater immune defense against the less virulent strain. IFI27 is mainly implicated in causing apoptosis, as would occur during a cytopathic infection. Both strains used in our study are, however, classified as being non-cytopathic, as supported by evidence from our measurements of cell viability which did not change following infection.

Although the primary action of IFNT during pregnancy recognition is directed at the endometrium, IFNT is also known to influence ISG expression in other tissues. Gifford et al. (80) observed that expression of some ISGs (*ISG15, MX1, MX2*) was up-regulated in peripheral blood leukocytes of pregnant dairy cows. They then showed that measurement of *ISG15* mRNA could provide a reliable indicator of early pregnancy on day 18 of gestation in heifers but not in mature cows (81). These findings have since been confirmed in beef breeds using both polymorphonuclear neutrophils and peripheral blood mononuclear cells (82, 83) and extending to a number of other ISGs (84). Although the results of *ISG15* up-regulation in early pregnancy were highly repeatable between studies, de Melo et al. (85) found that *ISG15* measurement was less reliable for confirmation of pregnancy than the use of circulating pregnancy-associated glycoprotein or Doppler ultrasound. This was due to more false positive and false negative results and the difference in response between heifers and cows. In light of our data, any future development of the use of ISG measurement in circulating leukocytes for pregnancy detection needs to be mindful of the ability of viral infections, including BVDV, to cause a similar response as this could potentially contribute to false positive tests.

## Conclusion

In bovine uterine endometrial cells, the IFNT challenge intensively stimulated expression of the 10 tested ISGs as expected. Inoculation with either high or low virulence strains of ncpBVDV-1 alone significantly inhibited the basal mRNA expression of eight of these ISGs (*GBP4, ISG15, HERC5, RSAD2, DDX58, IFI27, IFIT3*, and *MX1*) and increased expression of *TRIM56*. When the two treatments were combined, the presence of an ongoing BVDV infection generally suppressed the stimulatory effect of IFNT; for most of the tested genes, the effect was greater when the more virulent strain was used. In terms of immunity, these results suggest mechanisms whereby a more virulent strain of ncpBVDV-1 is better able to evade detection by host cells and to suppress antiviral responses. This could lead to greater systemic immunosuppression and enable the more virulent strain to proliferate more rapidly. Our results were limited to a cell culture system and endometrial transcription; however, they suggest potential mechanisms for the observed reductions in fertility in BVDV-infected cows. This could happen through a decreased ability of the endometrium to respond appropriately to either uterine bacterial infections after calving or to the pregnancy recognition factor IFNT.

## Data Availability Statement

The raw data supporting the conclusions of this article will be made available by the corresponding authors, without undue reservation.

## Ethics Statement

The animal study was reviewed and approved by the Royal Veterinary College ethical and Animal Welfare Committee and the United Kingdom Department for Environment, Food and Rural Affairs under the animal by-products regulation (EC) No. 1069/2009 (registration number U1268379/ABP/OTHER).

## Author Contributions

DCW, ZC, CT, and SZ designed the study. ZC and DCW wrote the manuscript. ZC, DCW, KW, CT, and SZ revised the manuscript. KW and ZC carried out the experiment. All authors contributed to the article and approved the submitted version.

## Conflict of Interest

The authors declare that the research was conducted in the absence of any commercial or financial relationships that could be construed as a potential conflict of interest.
