# Supplementary material for: Comparison of the Ability of High and Low Virulence Strains of Non-cytopathic Bovine Viral Diarrhea Virus-1 to Modulate Expression of Interferon Tau Stimulated Genes in Bovine Endometrium
Source: Front Vet Sci. 2021 Apr 9;8:659330. doi: 10.3389/fvets.2021.659330 (PMC8062762; doi:10.3389/fvets.2021.659330)
Supplement: Supplementary file 1 [file Data_Sheet_1.docx]

**Supplementary figure 1**. Effect of two ncpBVDV-1 strains, IFNT and their combination on the reference gene expression of (a) *ACTB*, (b) *GAPDH*, (c) *18SrRNA* and (d) *RPL10*. Primary cultures of mixed bovine endometrial cells (epithelium plus stroma) were cultured for 4 days before inoculation with either KY1203 (KY) or Ho916 (HO) strains of ncpBVDV-1. After a further 4 days, half the cultures were then stimulated with 100 ng/ml IFNT and the cultures were terminated 24 h later. CONT, untreated control cultures. All treatments were replicated using cells from 10 cows. Values are mean ± SEM, a>b>c, P<0.001.
