# Supplementary material for: Comparison of the Ability of High and Low Virulence Strains of Non-cytopathic Bovine Viral Diarrhea Virus-1 to Modulate Expression of Interferon Tau Stimulated Genes in Bovine Endometrium
Source: Front Vet Sci. 2021 Apr 9;8:659330. doi: 10.3389/fvets.2021.659330 (PMC8062762; doi:10.3389/fvets.2021.659330)
Supplement: Supplementary file 2 [file Data_Sheet_2.docx]

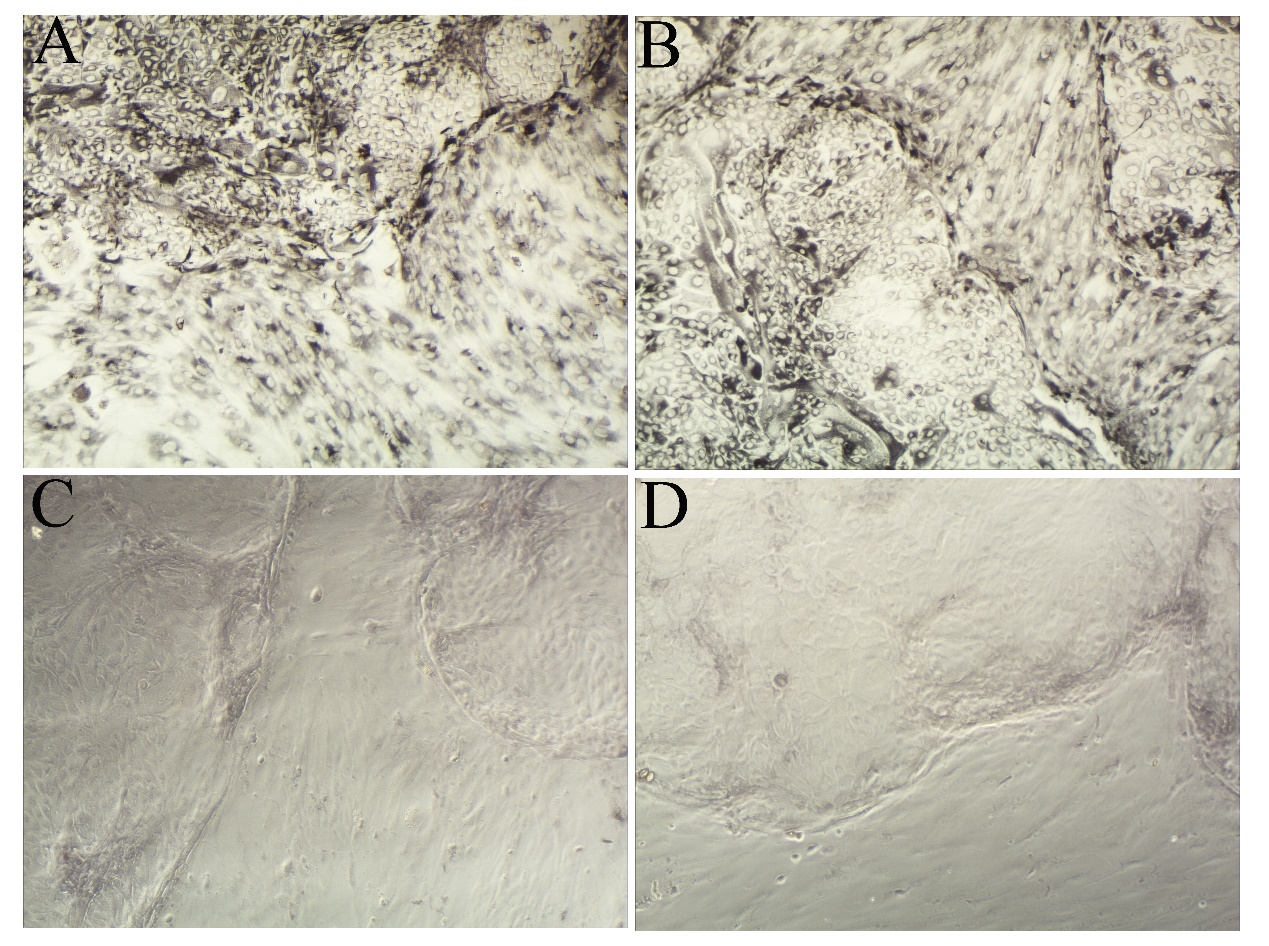


**Supplementary Figure 2.** Demonstration of infection of primary cultures of mixed bovine endometrial cells (epithelium plus stroma) with either (A) Ho916 (HO) or (B) KY1203 (KY) strains of ncpBVDV-1 using an indirect alkaline phosphatase immunostaining method. Dark cytoplasmic staining indicates that both epithelial and stromal cells were positive for BVDV. (C-D) BVDV was absent in the non-infected controls. Cells were cultured for 4 days before inoculation with each BVDV strain at a multiplicity of infection (MOI) of 0.1. The cultures were terminated 5 days later. All experimental work was undertaken from an early passaged virus stock. Bovine cells were used for all stages of the experimental work.
